# Neuronal Subtype and Satellite Cell Tropism Are Determinants of Varicella-Zoster Virus Virulence in Human Dorsal Root Ganglia Xenografts *In Vivo*

**DOI:** 10.1371/journal.ppat.1004989

**Published:** 2015-06-19

**Authors:** Leigh Zerboni, Ann Arvin

**Affiliations:** 1 Department of Pediatrics, Stanford University School of Medicine, Stanford, California, United States of America; 2 Departments of Pediatrics and Microbiology & Immunology, Stanford University School of Medicine, Stanford, California, United States of America; Louisiana State University Health Sciences Center, UNITED STATES

## Abstract

Varicella zoster virus (VZV), a human alphaherpesvirus, causes varicella during primary infection. VZV reactivation from neuronal latency may cause herpes zoster, post herpetic neuralgia (PHN) and other neurologic syndromes. To investigate VZV neuropathogenesis, we developed a model using human dorsal root ganglia (DRG) xenografts in immunodeficient (SCID) mice. The SCID DRG model provides an opportunity to examine characteristics of VZV infection that occur in the context of the specialized architecture of DRG, in which nerve cell bodies are ensheathed by satellite glial cells (SGC) which support neuronal homeostasis. We hypothesized that VZV exhibits neuron-subtype specific tropism and that VZV tropism for SGC contributes to VZV-related ganglionopathy. Based on quantitative analyses of viral and cell protein expression in DRG tissue sections, we demonstrated that, whereas DRG neurons had an immature neuronal phenotype prior to implantation, subtype heterogeneity was observed within 20 weeks and SGC retained the capacity to maintain neuronal homeostasis longterm. Profiling VZV protein expression in DRG neurons showed that VZV enters peripherin+ nociceptive and RT97+ mechanoreceptive neurons by both axonal transport and contiguous spread from SGC, but replication in RT97+ neurons is blocked. Restriction occurs even when the SGC surrounding the neuronal cell body were infected and after entry and ORF61 expression, but before IE62 or IE63 protein expression. Notably, although contiguous VZV spread with loss of SGC support would be predicted to affect survival of both nociceptive and mechanoreceptive neurons, RT97+ neurons showed selective loss relative to peripherin+ neurons at later times in DRG infection. Profiling cell factors that were upregulated in VZV-infected DRG indicated that VZV infection induced marked pro-inflammatory responses, as well as proteins of the interferon pathway and neuroprotective responses. These neuropathologic changes observed in sensory ganglia infected with VZV may help to explain the neurologic sequelae often associated with zoster and PHN.

## Author Summary

Varicella zoster virus (VZV) causes varicella; herpes zoster results from VZV reactivation and is associated with post herpetic neuralgia (PHN). We hypothesized that VZV exhibits neuron-subtype specific tropism and that VZV tropism for satellite glial cells (SGC) results in loss of SGC functions that support neurons and contributes to VZV-related ganglionopathy. Using human DRG xenografts in SCID mice, we demonstrated that initial VZV access to neuronal cell bodies occurs by the axonal route, followed by axonal and contiguous spread between neuron-satellite cell complexes. VZV replication is restricted in mechanoreceptive neurons compared to nociceptive neurons. Despite restricted infection, mechanoreceptive neurons were selectively depleted in association with SGC loss following acute DRG infection. VZV infection of DRG triggers release of pro-inflammatory cytokines that cause neuronal damage. These observations may help to explain the neurologic sequelae often associated with herpes zoster and PHN.

## Introduction

Varicella-zoster virus (VZV), a human alphaherpesvirus, causes varicella, characterized by a T cell-mediated viremia and a generalized vesicular rash [1]. During varicella infection, virions gain access to nerve cell bodies and establish latency, persisting for the life of the host. VZV reactivation from neuronal latency may cause herpes zoster, with pain and rash corresponding to the affected dermatome, and may be complicated by post herpetic neuraliga [2]. VZV reactivation can also produce chronic radicular pain without skin lesions (zoster sine herpete), cranial nerve palsies and other neurologic syndromes [2].

Investigating VZV neuropathogenesis is difficult because of its marked restriction for its natural human host. While VZV readily infects and replicates in neurons derived from embryonic stem cells and neuronal cell lines, these systems do not model the *in vivo* heterogeneity of DRG neuronal subpopulations and their associated satellite glial cells (SGC). DRG neurons derive from successive waves of migrating neural crest cells which differentiate into the two principal morphologic subtypes, which are “large, light” and “small, dark” neurons. Large light neurons comprise 30–40% of adult differentiated DRG neurons and have myelinated A beta-fibers with specialized mechanoreceptive termini in skin [3]. Small dark neurons with unmyelinated C-fibers or thinly myelinated A delta-fibers have “pain sensing” nociceptive free nerve endings in skin and comprise 40–60% of DRG neurons [4,5]. DRG are comprised of a structured tissue architecture in which individual ganglionic neurons are ensheathed by a single layer of SGC forming a neuron-satellite cell complex (NSC), which is a single functional unit in which SGC support neuronal homeostasis [6–8]. The neuronal surface has membrane protrusions that extend between laminar SGC [9], which are separated from the neuronal plasma membrane by a gap of 15–20 nm [8]. SGC participate in neuronal signaling, as well as pathological degeneration and regeneration of axons [8].

To examine VZV replication and spread within an intact ganglionic architecture, we developed a model using human dorsal root ganglion (DRG) xenografts in mice with severe combined immune deficiency (SCID) [10]. Intact DRG xenografts are maintained without rejection under the renal capsule of SCID mice (reviewed in ref. [11]). DRG xenografts contain clusters of heterogeneous nerve cell bodies, ensheathed by SGC, as well as axonal projections within their typical tissue microenvironments. This model provides an opportunity to examine viral determinants of VZV neurotropism, susceptibility of neuronal subtypes to infection and the characteristics of VZV-related polykaryon formation and spread between neurons and satellite cells during VZV replication in sensory ganglia *in vivo*, in the absence of VZV-specific adaptive immunity. These conditions could occur for a limited period during primary infection and may account for the occasional appearance of a zoster-like rash during varicella in the healthy host, and could also occur during the few days following reactivation, or perhaps longer in elderly and immunocompromised patients who have low frequencies of VZV-specific responder T cells and a slow expansion and mobilization of an effective adaptive immune response to the affected ganglion.

Our previous work using the DRG model established that even though both VZV and herpes simplex virus (HSV)-1 infect sensory ganglia, VZV replicates in SGC and can induce cell-cell fusion between SGC and neurons in neuron-satellite cell complexes (SCC) [12], whereas HSV-1 does not [13]. Neuron-satellite cell fusion is also observed in ganglia from patients with zoster at the time of death, indicating that SGC tropism is a component of VZV neurovirulence [14]. To further investigate VZV neuropathogenesis, we hypothesized that VZV would exhibit specificity for neuronal subtypes and that the capacity to infect SGC would facilitate VZV spread in DRG and contribute to VZV-related ganglionopathy. Here, we further characterized the differentiation of neurons in DRG xenografts and show that VZV replication is restricted in RT97-immunoreactive mechanoreceptive neurons compared to peripherin-positive nociceptive neurons, based on patterns of VZV protein expression and intracellular localization. In addition, the susceptibility of SGC to VZV replication is implicated as a factor in neuronal cell loss. Finally, we document changes in the DRG tissue milieu due to the induction of cellular factors with antiviral and neuroprotective functions as well as marked upregulation of proinflammatory proteins associated with neuronal damage. Our observations identify neuropathologic changes in sensory ganglia due to VZV infection that help to account for the long lasting neurologic sequelae often associated with herpes zoster or zoster sine herpete.

## Results

### Differentiation of neurons after DRG implantation

**Fig 1:**
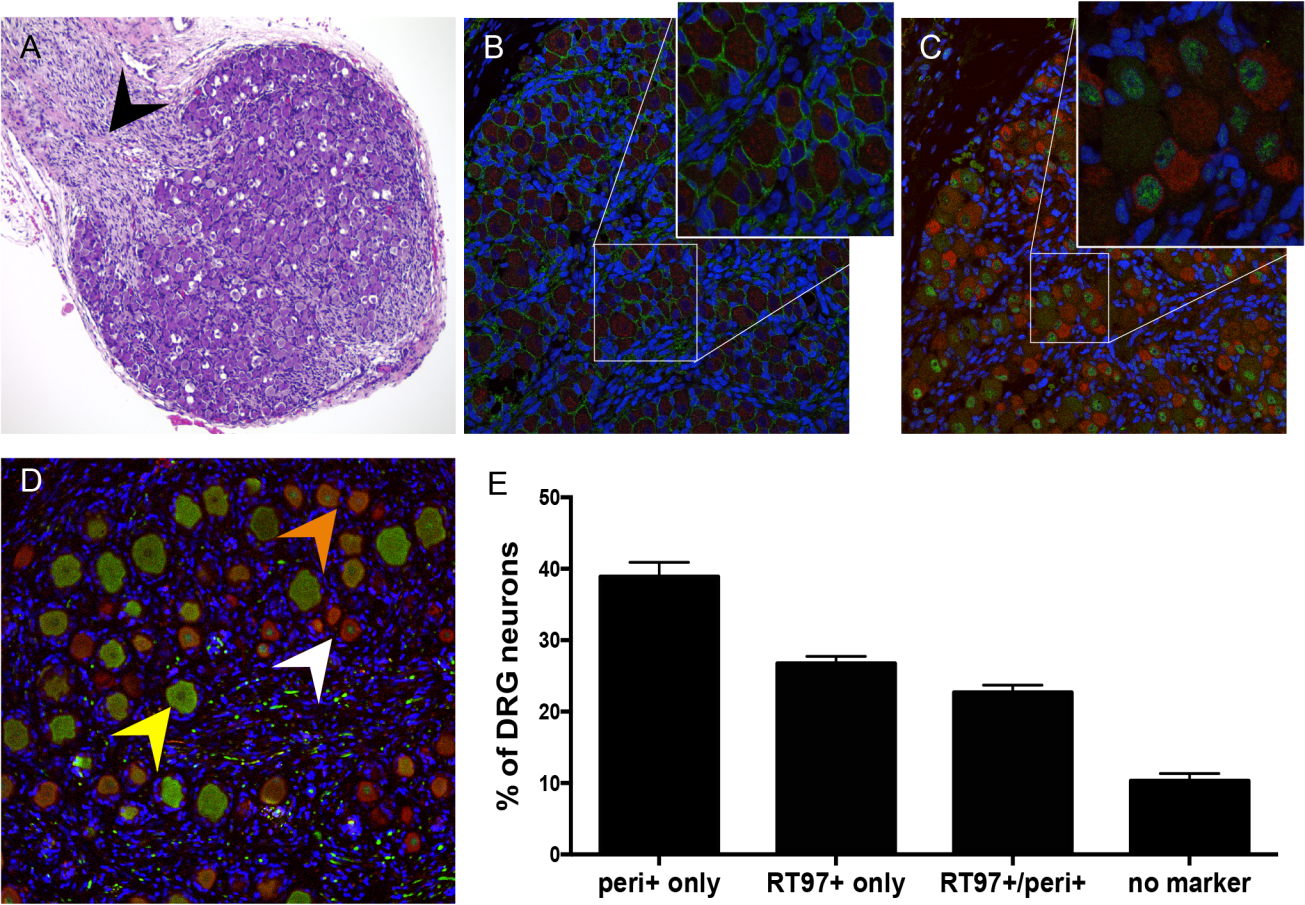
Differentiation of neurons after DRG implantation. Panels A-C, fetal DRG, 18 gestational weeks, prior to subcapsular renal implantation in SCID mouse, stained with (A) hematoxylin & eosin; black arrow denotes nerve root, (B) anti-NCAM (green)/anti-synaptophysin (red) antibody, and (C) subtype specific markers anti-RT97 (green)/anti-peripherin (red) antibody. Panels D-E, DRG xenograft, 20 weeks after transplantation, stained with subtype specific markers anti-RT97 (green)/anti-peripherin (red) antibody (D), demonstrates that neuronal differentiation continues in DRG xengrafts *in vivo*. Peripherin staining (white arrow) identifies nociceptive neurons; cytoplasmic RT97 immunoreactivity (yellow arrow) identifies mechanoreceptive neurons. Cell counting (E) determined the proportion of nociceptive and RT97-immunoreactive mechanoreceptive neurons at 20 weeks after implantation. For each panel, representative images are shown. For assessment of neuronal subtype at least 20 fields were counted, each with a minimum of 50 neurons.

To investigate the subtype specificity of VZV neurotropism, we first evaluated neuronal differentiation in DRG xenografts before transplantation, at 18 gestational weeks, and at 20 weeks after transplantation. At 18 weeks gestation, by which time major events in human sensory neurogenesis are complete, fetal DRG were densely packed with sensory neurons and nerve fibers extending from the dorsal root (Fig 1A, black arrow). All DRG neurons expressed the pan-neuronal cell markers, neural cell adhesion molecule (N-CAM) and synaptophysin (Fig 1B) in their expected membrane and cytoplasmic patterns, respectively. Whereas both mature and immature nociceptive neurons express the cytoplasmic neurofilament subunit peripherin [15], differentiated mechanoreceptive neurons acquire cytoplasmic expression of the 200 kDa neurofilament (NF200) subunit, which has a phosphoepitope recognized by the RT97 antibody [16]; this marker is present in all neuronal nuclei during growth phase [17]. Prior to DRG implantation, RT97 immunoreactivity was restricted to neuronal nuclei and cytoplasmic immunoreactivity was absent, indicating an immature neuronal immunophenotype (Fig 1C); however, by 20 weeks after implantation, mature mechanoreceptive neurons (Fig 1D, yellow arrow) were readily identified by cytoplasmic RT97 immunoreactivity. As expected, the neurofilament subunit peripherin was detected in the cytoplasm of small-diameter neurons only (Fig 1D, white arrow). Quantitative neuronal immunophenotyping demonstrated that 38.9% of neurons were nociceptive (peripherin-positive) and 26.7% were mechanoreceptive neurons (cytoplasmic RT97-positive) at 20 weeks after implantation (Fig 1E). 22.7% of DRG neurons were dual RT97-peripherin positive (Fig 1D, orange arrow) and 10.3% did not express either marker (Fig 1E). Of note, adult ganglia have subpopulations of dual RT97+/peripherin+ neurons [3,4]. Thus, continued neuronal differentiation in DRG xenografts resulted in proportions of neuronal subtypes found in postnatal and adult human ganglia [5,18,19].

### DRG architecture is maintained in DRG xenografts

**Fig 2:**
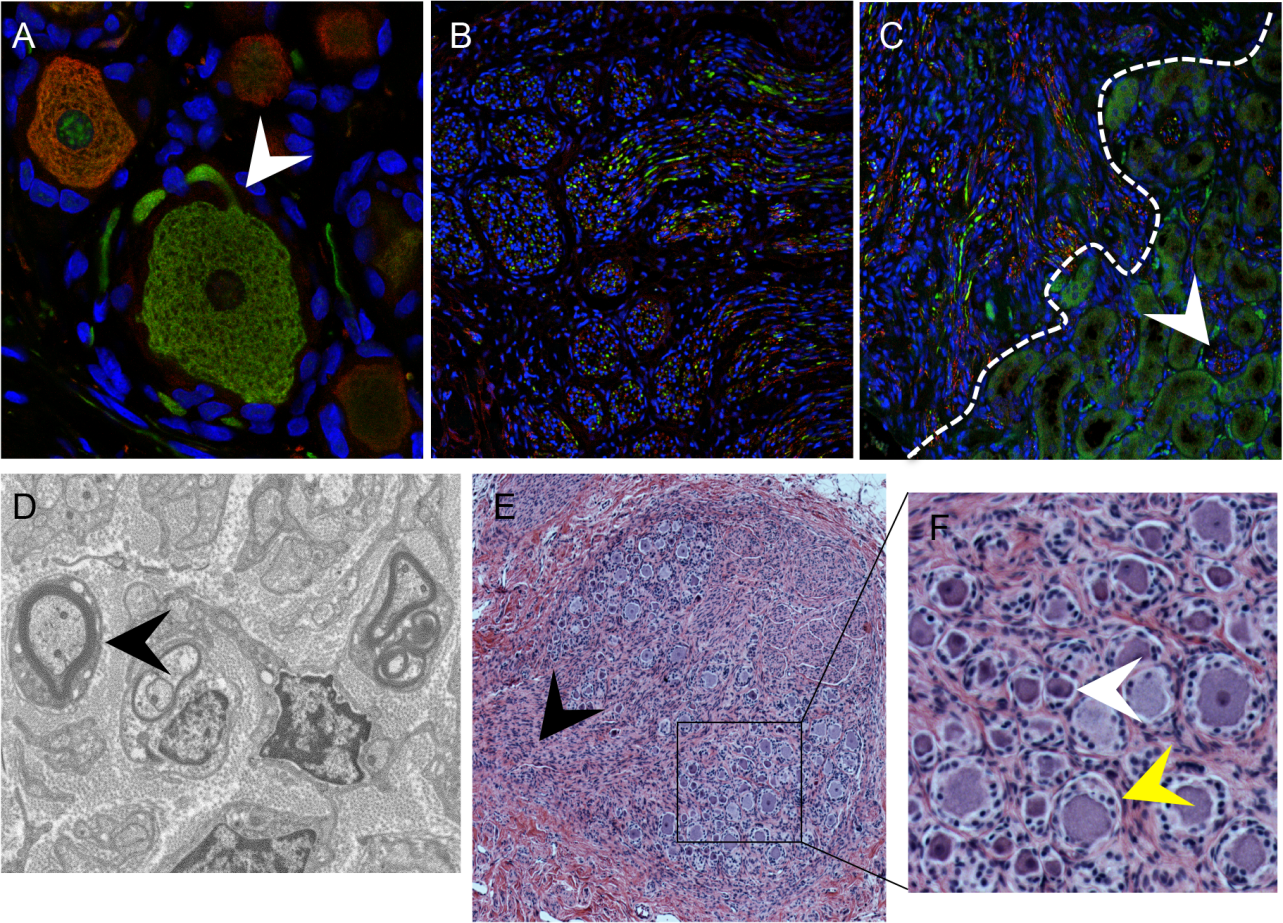
DRG architecture is maintained in DRG xenografts. Panels A-C, DRG xenograft, 20 weeks after transplantation, stained with subtype specific markers anti-RT97 (green)/anti-peripherin (red) antibody (A-C). White arrow in (A) denotes the axon hillock at the neuronal cell body. White dotted line in (C) delineates the margin between the DRG xenograft and the murine kidney, arrow shows axons projecting into the murine kidney. Panel D, transmission electron micrograph of DRG xenograft with arrow showing myelinated nerve fiber. Panel E, DRG xenograft 80 weeks after implantation. Black arrow in (E) denotes the nerve root, panel on the right (F) is inset panel (black box) from (E) with arrows showing small, dark neurons (white) and large, light neurons (yellow).

The typical DRG tissue architecture was confirmed at 20 weeks after implantation by the presence of neuronal structures such as the axon hillock, the junction between the nerve cell body and the projecting axon (Fig 2A, white arrow), and nerve bundles containing both mechanoreceptive (RT97+) and nociceptive (peripherin+) nerve fibers extending throughout the DRG xenograft (Fig 2B) as well as into the murine renal tubule network (Fig 2C, white arrow). The survival of neurons and axons within the xenografts and extensions into the murine kidney indicated that the growth properties of differentiated DRG neurons were intact despite absence of orthotopic synaptic partners. These properties also indicated that the capacity of SGC to maintain neuronal homeostasis by producing neurotrophins, including NGF and other factors, was preserved [20]. Neurotrophins secreted in murine nephrons may act as axon guidance cues [21,22]. The presence of thick myelin-wrapped nerve fibers was demonstrated by transmission electron microscopy (Fig 2D, black arrow). DRG xenografts maintained a mature DRG architecture for at least 80 weeks after implantation, with comparative proportions of RT97 and peripherin positive neurons as observed at 20 weeks after transplantation, and including physical features such as a visible dorsal root (Fig 2E, black arrow), axon bundles and clusters of small, dark-appearing (Fig 2F, inset, white arrow) and large, light-appearing (Fig 2F, inset, yellow arrow) nerve cell bodies.

### VZV access to nerve cell bodies in DRG occurs by axonal transport and by contiguous spread from infected SGC

**Fig 3:**
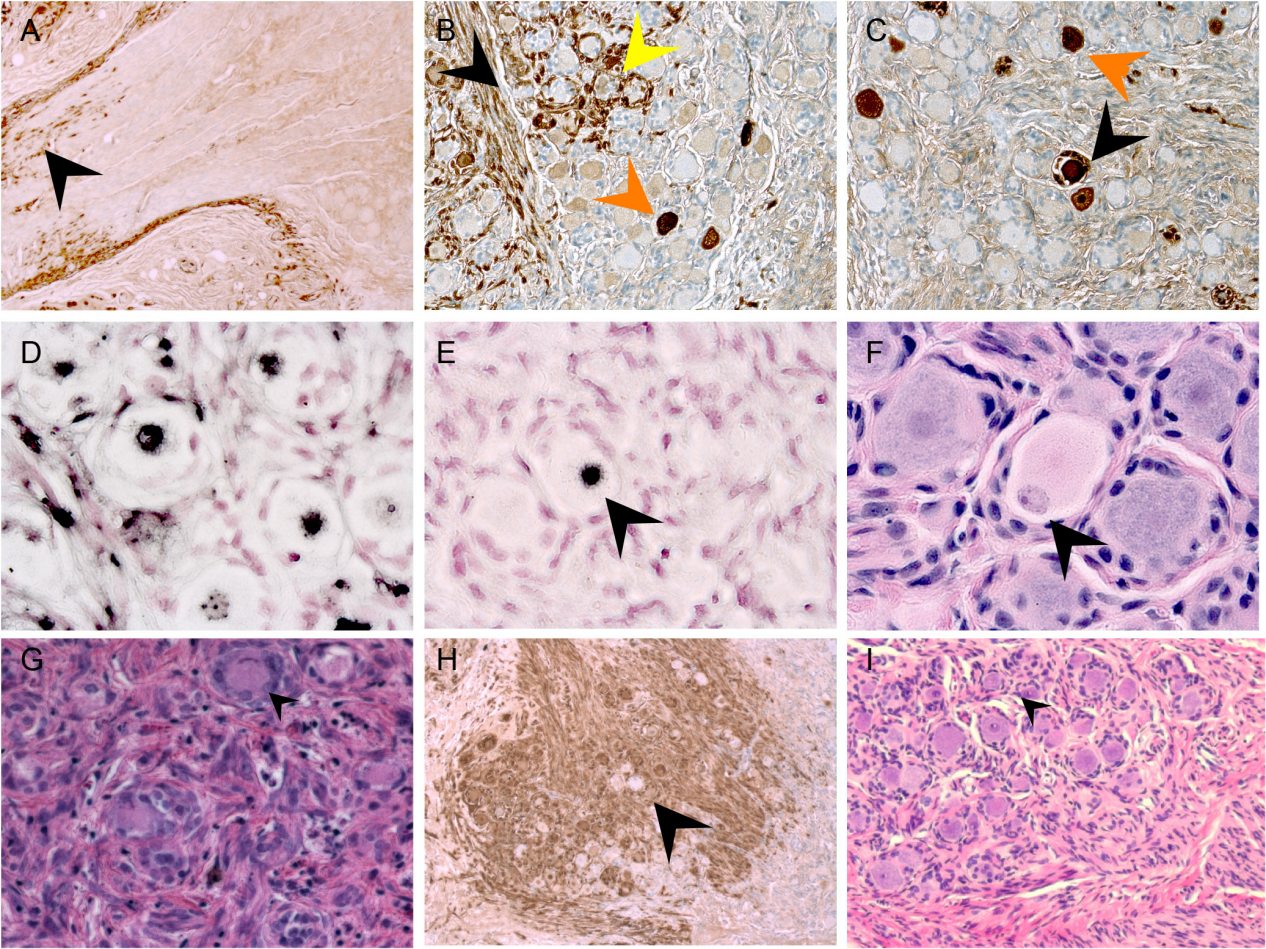
VZV spread in DRG xenografts. VZV-infected DRG xenograft immunostaining at 7 (A), 10 (B-F) and 14 days (G-H) after infection. Representative images are shown for staining experiments, performed on multiple tissue sections (6–10 sections) for each DRG, to assess cytopathology by VZV IE63 protein (A-C, and H), H&E stain (F, G and I), and VZV genomic DNA (D and E). Black arrow in A,B shows IE63 protein in nerve fibers; orange arrow in B,C shows IE63 protein in neuronal cell; yellow arrow in B shows IE63 staining in SGC. Arrow in E,F shows adjacent tissue sections stained for VZV DNA (E) and H&E (F), demonstrating neuronal chromatolysis. Arrow in G shows regions with extensive cytopathic effect; arrow in H shows IE63 negative neuron at 14 days after infection.

In previous work, we demonstrated efficient replication of VZV (recombinant parent Oka strain) in DRG xenografts at 4–12 weeks after implantation, peaking at 14 days after inoculation [10,23]. To assess VZV infection during the earliest stages of spread in differentiated neurons, we examined DRG xenografts inoculated at 25 weeks with recombinant parent Oka strain VZV-infected fibroblasts exhibiting high cytopathic effect which results in low titer (330 PFU/implant; 10 microliters). DRG xenografts were recovered at 3, 7, and 10 days as well as at 14 days after infection [10,23]. Patterns of spread were identified by analyzing IE63 expression and VZV genomic DNA in sequential tissue sections containing clusters of nerve cell bodies that exhibited cytopathic effects. No VZV protein or viral genomes were detected in either neurons or SGC at three days after inoculation and the DRG tissue architecture showed no disruption, indicating that inoculum cells are not identified in the DRG and that an eclipse phase occurs during which secondary replication remains below the threshold of detection. At day 7, IE63 was detected within nerve fibers (Fig 3A, black arrow), along the nerve root, and in regions containing neuronal cell bodies (Fig 3B, black arrow). In some regions, IE63 expression was observed in neurons but not in their surrounding SGC (Fig 3B and 3C, orange arrow), or in non-neuronal supportive cells, showing that neuronal infection was not due to contiguous spread. Staining of adjacent tissue sections did not reveal any IE63 expression in neighboring SGC, indicating that initial VZV spread to neuronal cell bodies may occur axonally following virion entry at axon terminal sites within DRG xenografts. Neurons expressing IE63 were also observed with IE63 staining in the SGC encapsulating the neuron (Fig 3C, black arrow). In addition, IE63 positive SGC were observed surrounding IE63 negative neurons (Fig 3B, yellow arrow), indicating that VZV may spread between contiguous NSC by infection of SGC. Examination of VZV DNA localization showed the same patterns as IE63 expression (Fig 3D and 3E), providing further evidence for both axonal transfer and contiguous spread from SGG into neurons. Notably, VZV DNA-positive neurons exhibited characteristics of axonal neuropathy, such as chromatolysis (Fig 3E and 3F, arrow), a cell body reaction that indicates activation of axonal repair processes [24]. By day 14 after infection, neuronal and SGC cytopathic effects were extensive (Fig 3G). SGC infection allows secondary infection of SGC in the adjacent NSC, enabling viral access to another neuronal cell body within the ganglion.

### Restriction of VZV replication in mechanoreceptive neurons

**Fig 4:**
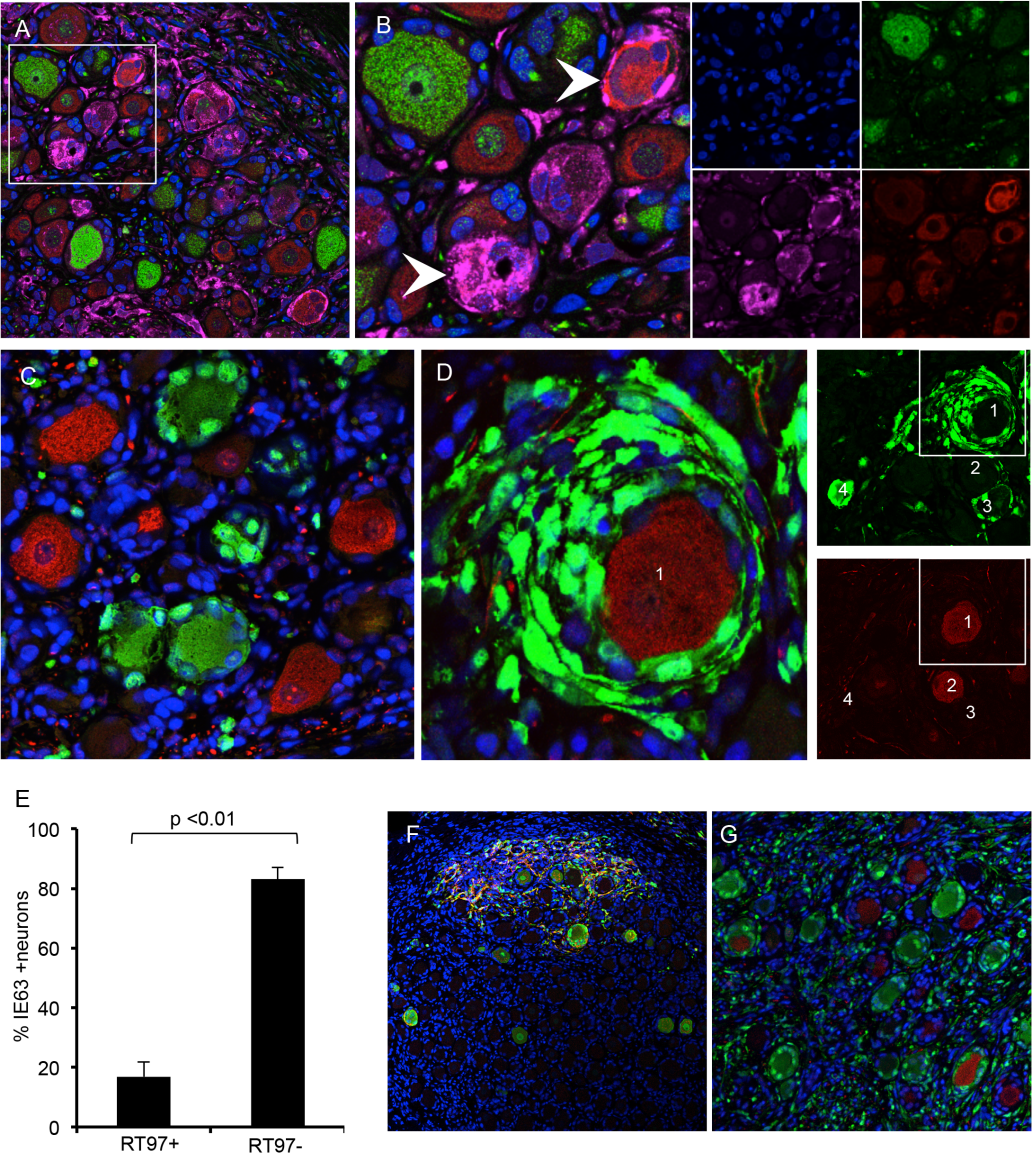
VZV replication is restricted in mechanoreceptive neurons. Immunostaining VZV-infected DRG xenograft 7–10 days after infection with 330 PFU. Staining experiments were performed on multiple tissue sections (6–10 sections) for each DRG. Panel A, DRG tissue section stained for RT97 marker (green), peripherin (red) and anti-VZV human polyclonal antibody (pink); (B) is inset white panel from A with single channel images to the right. To quantify IE63 positive/RT97 positive neurons compared with IE63 positive/RT97 negative neurons; a minimum of 250 neurons was counted for each condition. Panel C, representative image for cell counting analysis, demonstrating IE63 restriction (green) in RT97 positive (red) neurons (t test p<0.01). (D) Representative image showing extensive IE63 staining (green) in SGC surrounding an RT97-immunoreactive (red) neuron. Single channel images at lower magnification (right) show four numbered neurons in a region with IE63 expression (top) in satellite cells surrounding RT97+ (red) neuron 1 and neuron 3. (E) Cell counting results. (F-G) DRG inoculated with VZV infected T cells (1067 PFU) exhibit the same patterns of spread in xenografts and restricted replication in RT97 immunoreactive neurons. Sections are stained for IE63 (green), and gE (Panel F, red) and RT97 (Panel G, red).

Because some neurons with large diameter morphology did not exhibit cytopathic changes (Fig 3G, black arrow) or express IE63 (Fig 3H, black arrow), even where DRG infection was widespread, we next analyzed expression of VZV proteins in sections stained for peripherin and the RT97 marker (Fig 4A and 4B). When using an anti-VZV human polyclonal antibody, peripherin/VZV dual positive neurons were readily identified (Fig 4B, white arrows). We then examined IE63 protein expression in neurons that were positive or negative for the RT97 marker of mature mechanoreceptive neurons. More than 250 neurons were examined in tissue sections from three DRG recovered 7–14 days after infection; a representative image is shown (Fig 4C). Overall, 41.3% of all neurons were RT97 positive and 36.5% were IE63 positive. Of the IE63 positive neurons, only 16.9% were RT97 positive, whereas 83.1% were RT97 negative (Fig 4E, p<0.0001, t test). The proportion of neurons expressing RT97 in infected and uninfected DRG was equivalent, indicating that the difference did not reflect down regulation of the RT97 marker by VZV. These results indicated that RT97+ neurons have a restricted capacity to support lytic VZV infection. Notably, neurons that were IE63 negative despite being encapsulated by IE63 positive SGC consistently belonged to the RT97 subpopulation (Fig 4D).

During primary infection, VZV reaches ganglia by retrograde axonal transport from nerve endings in skin lesions and hematogenously through transport by infected T cells (reviewed in ref. [11]). Unlike cell-cell fusion and syncytia formation observed in cultured cells and skin, VZV-infected T cells release virus particles but do not undergo fusion [25]. To determine if the characteristics of VZV spread and subtype specific restriction might be an artifact of some capacity of infected fibroblasts to initiate VZV transfer by cell-cell fusion, which would not occur during natural infection, DRG xenografts were inoculated with VZV-infected T cells (1067 PFU/implant) and examined at 14 days after infection. The same patterns of axonal and contiguous VZV spread were observed (Fig 4F) and again infection was restricted within the RT97 positive subpopulation after T cell transfer (Fig 4G).

### Restriction of VZV replication in mechanoreceptive neurons occurs after entry

**Fig 5:**
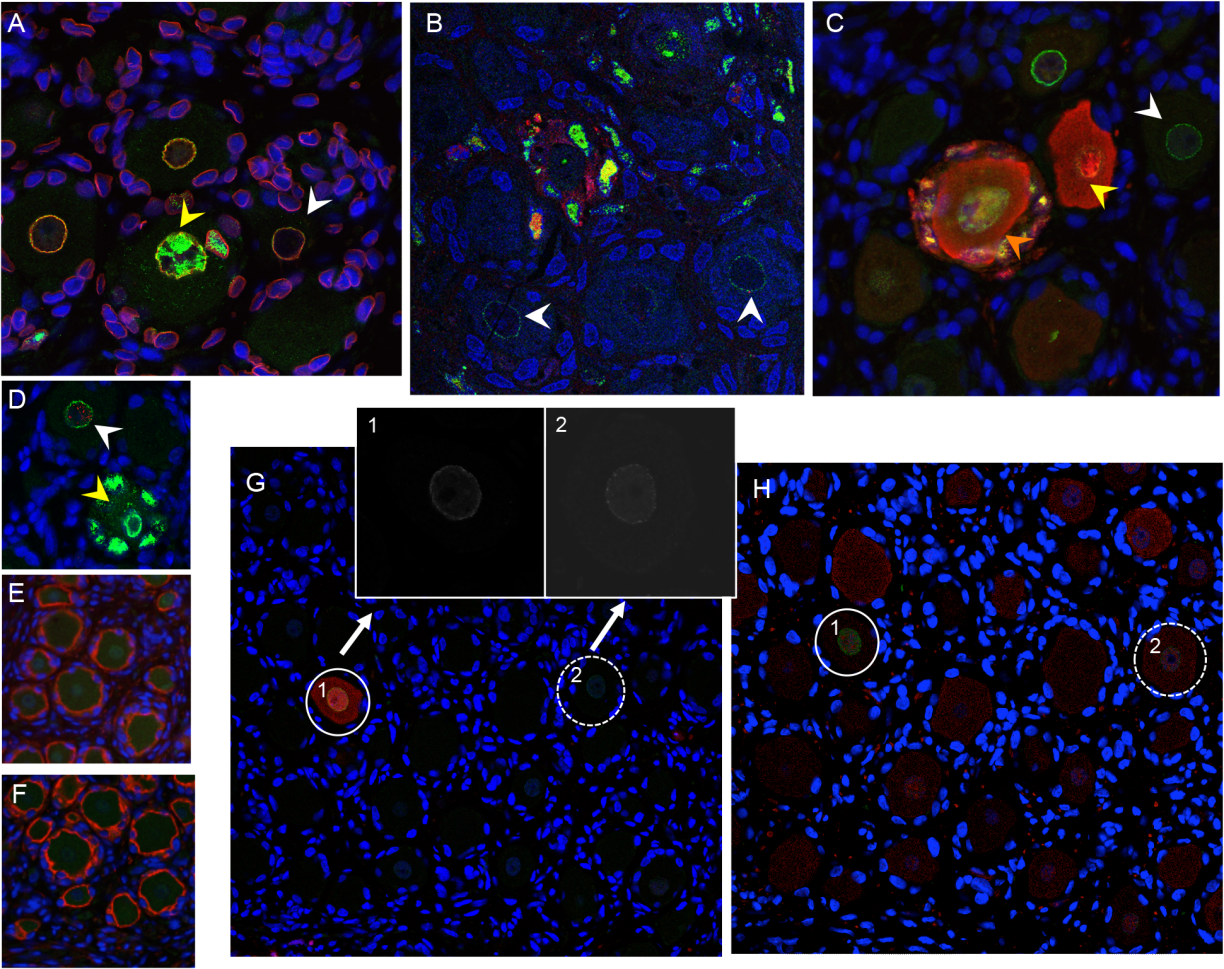
VZV restriction in mechanoreceptive neurons occurs after viral entry. For each panel, one representative image is shown for staining experiments performed on multiple tissue sections (6–10 sections) for each DRG. (A-C) ORF23 capsid protein is a marker of virion entry as well as the later formation of progeny virions; virion entry is indicated by ORF23 capsid puncta at the nuclear rim (green), co-stained with cellular lamin A/C (A, red), and prior to expression of IE62 (B, red) or IE63 (C, red). (D) Dual staining for ORF23 (green) and cellular PML (red). (E) ORF23 nuclear rim staining was not observed when using pre-immune serum (green); red is N-CAM staining. (F) ORF23 capsid protein is not detected in uninfected DRG, as shown by staining for N-CAM (red) and absence of ORF23 (green). (G-H) Staining of adjacent tissue sections stained with antibody for ORF23 (green) and IE63 (G, red) or RT97 (H, red). Two neurons are circled and shown in both panels. The single 488-channel images for Panel G, shown in greyscale for better visualization, are provided for the circled neurons (1 and 2). The contrast of the G, neuron 2, ORF23 B&W panel, is enhanced so that the discrete ORF23 particles are easier to observe.

Expression of the ORF23 capsid protein and the ORF61 and IE62 viral regulatory proteins, which are made first and before IE63 in newly infected cells *in vitro* [26], was used to define the block of VZV infection in RT97+ neurons. ORF23 capsid protein expression in nuclear puncta marks the incoming virions in cell culture [26]. In DRG, ORF23 appeared in rare clusters of neurons at 7 days after infection as discrete dim puncta at the nuclear rim, shown by co-staining for nuclear lamins (Fig 5A, white arrow). ORF23 puncta formed a nearly complete ring in some neurons, without IE62 (Fig 5B, white arrows) or IE63 (Fig 5C, white arrow). In other neurons, ORF23 expression was intense along a crenellated nuclear rim, often accompanied by reorganization of promyelocytic leukemia protein (PML) nuclear bodies (Fig 5D, white arrow) at 7–10 days. PML nuclear bodies are first reorganized and later dispersed as VZV infection progresses (Fig 5D, yellow arrow) [26,27]. Crenelated nuclear rim expression and ORF23 expression in globular domains was accompanied by IE63 expression (Fig 5A and 5C, yellow arrows). Both of these patterns indicate later stages of VZV infection. Controls including infected DRG tested with preimmune rabbit anti-ORF23 serum (Fig 5E) and uninfected DRG tested with anti-ORF23 were negative (Fig 5F). ORF23 nuclear rim staining did not differ in neurons in relation to their expression of the RT97 marker, showing entry had occurred. In the representative example shown (Fig 5G and 5H), discrete ORF23 nuclear rim staining was observed in both IE63+/RT97 negative (Fig 5G and 5H, inset box G1) and IE63-/RT97+ neurons (Fig 5G and 5H, inset box G2).

### Restriction of VZV replication in mechanoreceptive neurons occurs after ORF61 and before IE62 expression

**Fig 6:**
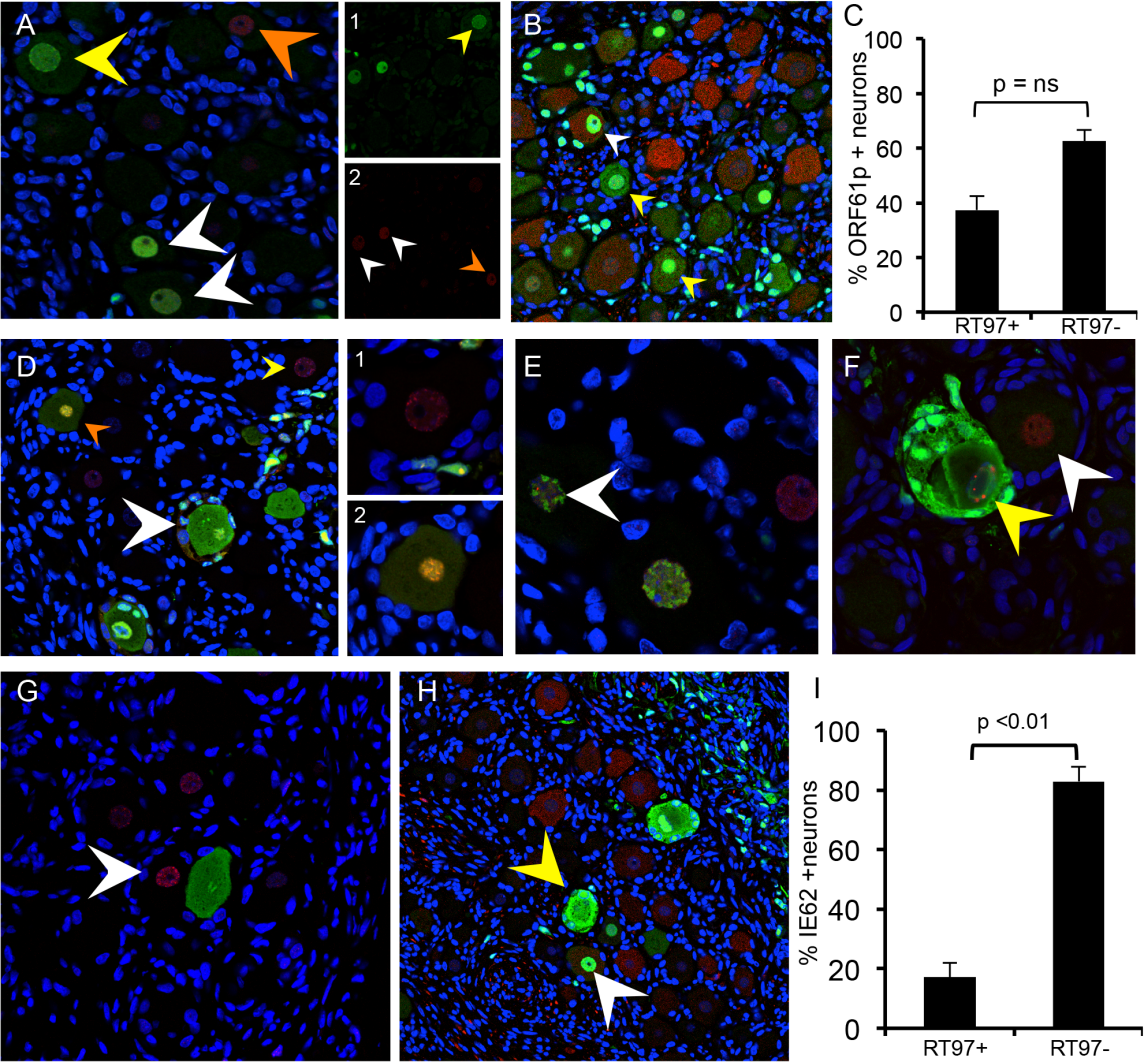
Restriction of VZV replication in mechanoreceptive neurons is associated with restricted IE62 expression. As described in the Results, reorganization and disruption of PML nuclear bodies were assessed in relation to expression of VZV ORF61 (A, green), IE62 (E and F, green) and IE63 (G, green); PML staining is in red. Representative images are shown; arrows are described in the text. (D) Temporal expression of IE62 (red) and IE63 (green) protein in the same neuron cell body; (D, right) shows the two neurons in panel (D) at higher magnification. (B,C and H,I) Cell counting analysis of ORF61p (C) and IE62 (I) expression in relation to the RT97 subtype marker. Representative panels are shown for ORF61 protein (B, green) and IE62 (H, green) and RT97 marker (B and H, red).

ORF61 is critical to disrupt PML nuclear bodies in skin *in vivo* [27]. In DRG, ORF61 was localized to neuronal nuclei, as occurs shortly after VZV entry (Fig 6A) [26] and limited ORF61 expression was associated with dim and diffuse PML detection in neurons (Fig 6A, white arrows). In contrast, PML nuclear bodies were dispersed when ORF61 expression was increased and present in both the nuclei and cytoplasm of infected neurons (Fig 6A, yellow arrow). PML nuclear bodies were also dim and diffuse in the nuclei of uninfected neurons within VZV-infected DRG (Fig 6A, orange arrow and Fig 6F, white arrow). When ORF61 expression was evaluated along with the RT97 marker, 37.4% of ORF61 positive neurons were RT97 positive and 62.6% were RT97 negative (p = ns, t test) (Fig 6B: representative image). However, ORF61 was predominantly nuclear in RT97 positive neurons (Fig 6B, white arrow) whereas RT97 negative neurons had cytoplasmic ORF61 expression (Fig 6B, yellow arrows).

IE62 is the major viral transactivator and functions to block the interferon (IFN) pathway in VZV-infected cells [28,29]. At 7–10 days after infection, IE62 appeared in discrete puncta along the nuclear rim (Fig 6D, yellow arrow and box D1 on right), in the absence of IE63. IE62 expression was more intense within larger intranuclear domains in DRG neurons when diffuse and faint IE63 nuclear expression became detectable, indicating progression of infection (Fig 6D, orange arrow and box D2 on right). The formation of IE62-positive intranuclear domains was associated with reorganized PML although IE62 did not completely co-localize with PML nuclear bodies (Fig 6E, white arrow), consistent with observations *in vitro*. PML were reorganized prior to IE63 expression (Fig 6G, white arrow). Cytoplasmic IE62 was extensive in neurons with markers of late infection, including plasma membrane dissolution and fusion of neurons and their encapsulating SGC (Fig 6D, white arrow), and the presence of large PML nuclear bodies that sequester VZV nucleocapsids and inhibit viral replication (Fig 6F, yellow arrow) [30]. Notably, only 17.1% of the IE62 positive neurons were RT97 positive and 82.9% were RT97 negative (p<0.0002, t test) (Fig 6H; representative image). When IE62 expression was observed in RT97 positive neurons, it was predominantly nuclear; IE62 cytoplasmic expression was rare in this subpopulation (Fig 6H, yellow arrows). Thus, although ORF61 was produced regardless of neuronal subtype, the restriction of VZV replication in RT97+ neurons was associated with low or no IE62 expression, which would impair progression to lytic replication.

### Selective depletion of mechanoreceptive neurons after acute DRG infection

**Fig 7:**
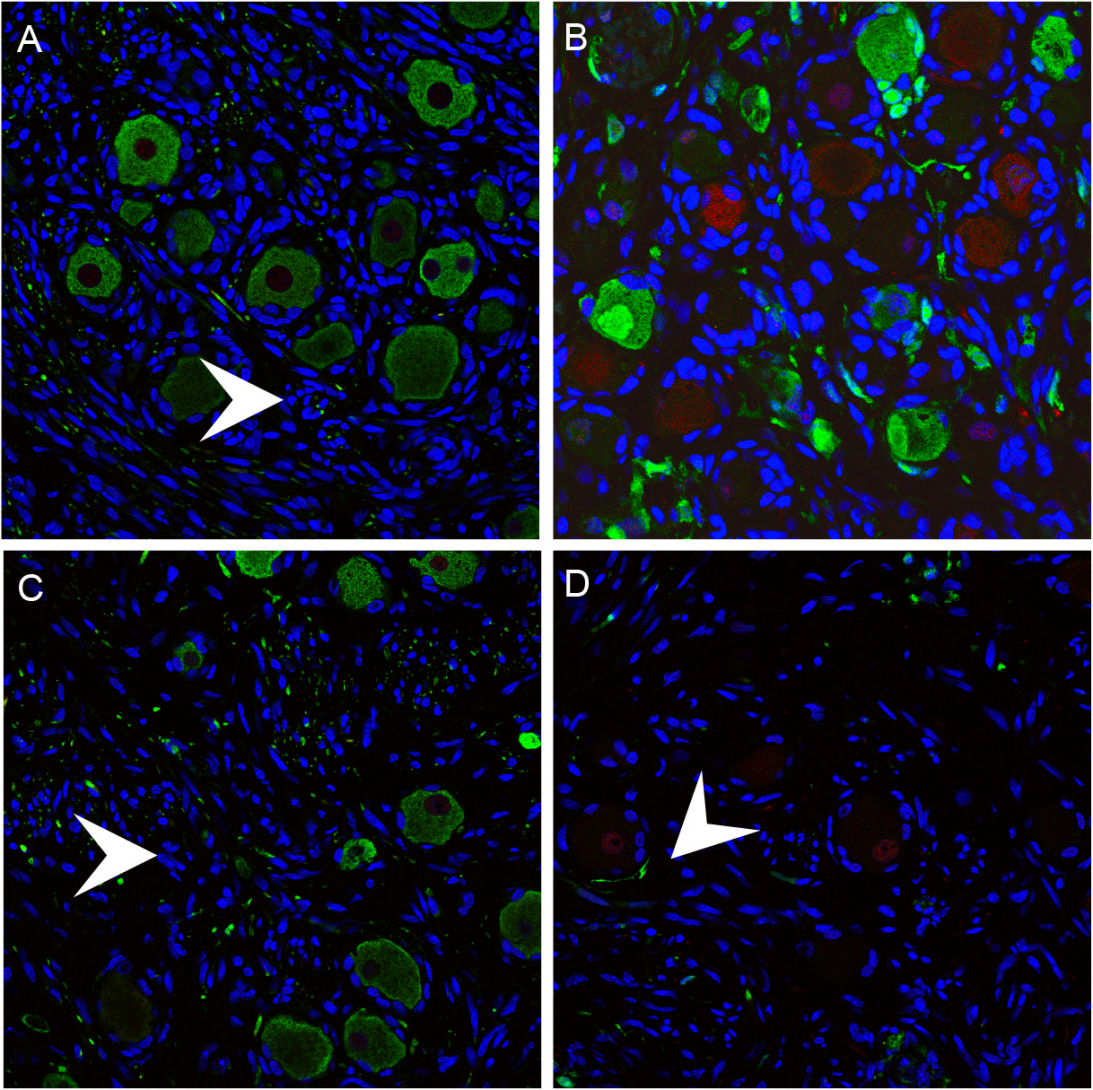
Mechanoreceptive neurons are selectively depleted after the acute phase of DRG infection and SGC infection contributes to VZV-induced neuronal cell loss and ganglionic damage. DRG xenografts were infected with rOka-delta_gI or rOka-gE/deltaCys (1000 PFU), which are deficient for VZV-induced SGC-neuron membrane fusion and spread in DRG, to assess the impact of infection limited primarily to SGC on neuronal survival. Representative images are shown; for each staining condition 6–10 slides were evaluated. (A) rOka-infected DRG at 70 days after infection, stained for subtype markers peripherin (green) and RT97 (red). White arrows indicate Nageotte nodules. (B) rOka-gE/deltaCys infected DRG at 28 days after infection, stained with mouse anti-RT97 (red) and rabbit anti-IE63 (green) and at 56 days after infection stained with mouse anti-RT97 (red) and rabbit anti-peripherin (green). (C) rOka-gE/deltaCys infected DRG at 56 days after infection stained with mouse anti-RT97 (red) and rabbit anti-IE63 (green). (D) rOka-delta_gI infected DRG at 70 days after infection stained with mouse anti-RT97 (red) and rabbit anti-IE63 (green).

By 70 days after infection, VZV proteins and infectious virions are no longer produced in DRG xenografts but low copies of viral genomes persist, similar to latently infected human cadaver ganglia [10]. Whereas uninfected DRG retained a normal tissue architecture over this period (Fig 2E), VZV-infected DRG exhibited fibrotic areas and clusters of SGC proliferations, referred to as Nageotte nodules, which mark sites of neuronal disappearance (Fig 3I, black arrow) [24]. Notably, even though VZV infection was restricted in RT97+ neurons, most neurons present at this late stage were peripherin positive while RT97+ neurons were almost entirely absent (Fig 7A), and Nageotte nodules were abundant (Fig 7A, white arrows). Only 3% of neurons were RT97 positive while 79% were peripherin-positive, demonstrating a significant loss of the mechanoreceptive subtype. The remainder expressed both peripherin and RT97 or neither marker, indicating a non-mechanoreceptive phenotype.

### Contribution of SGC infection to neuronal cell loss and ganglionic damage

Two VZV mutants, rOka-delta_gI, with a complete gI deletion, and rOka-gE/deltaCys, with a mutation blocking gE/gI heterodimer formation, infect SGC but are deficient for VZV-induced SGC-neuron membrane fusion and had little spread in DRG, as previously described [23]. Thus, infecting DRG with these mutants made it possible to evaluate effects on infection limited primarily to the contributions of SGC to neuronal survival. DRG infected with rOka-gE/deltaCys showed restricted IE63 expression in RT97 positive neurons at 28 days (Fig 7B) with selective loss of neurons of the RT97 subtype at day 56, and the appearance of Nageotte nodules (Fig 7C, white arrow). Similarly, IE63 was primarily limited to SGC and non-neuronal cells in rOka-delta_gI infected DRG (Fig 7D, white arrow). Neuronal cell loss was observed and was selective for RT97+ neurons, further indicating that SGC infection leads to neuronal loss and the RT97+ neurons are more susceptible to the neuropathic effects of ganglionic infection.

### Induction of cellular factors in VZV-infected DRG

**Fig 8:**
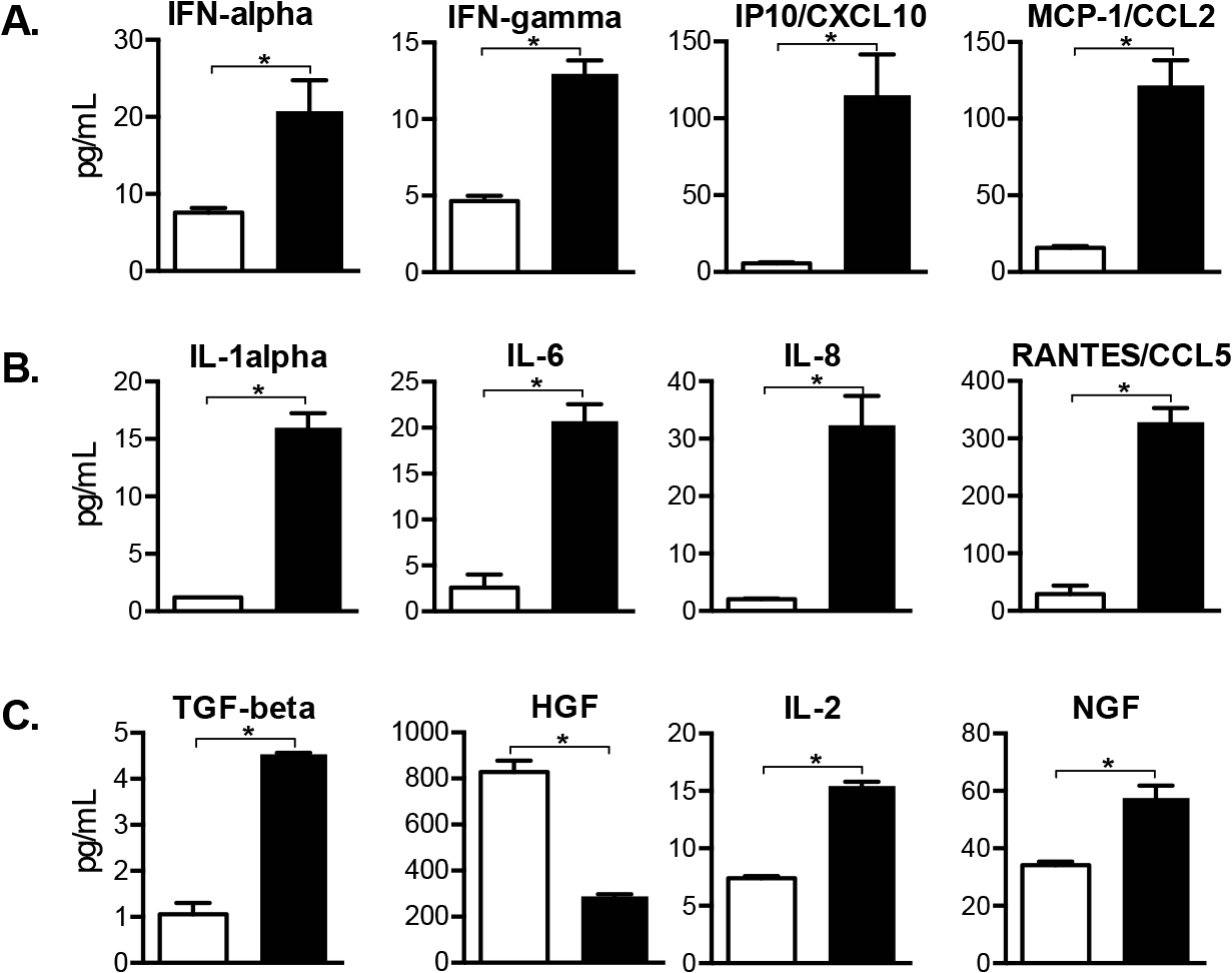
Cellular factors induced in VZV-infected DRG include antiviral and pro-inflammatory cytokines. Cellular factors made by SGC and other DRG resident cells were profiled in whole tissue lysates. Cytokine concentrations of 19 human proteins were significantly increased (N = 18) or decreased (N = 1) in VZV-infected DRG compared with mock-infected DRG (p <0.05), and 12 are grouped by role: (A) interferon-regulated cytokines, (B) pro-inflammatory cytokines and (C) neuroprotective cytokines. Statistical analyses were performed using GraphPad Prism version 6.0. The complete dataset is shown in S1 and S2 Figs.

Cellular factors produced by SGC regulate homeostasis as well as having macrophage-like functions [8] and neurons have intrinsic responses to stress. In these experiments, proteins made by DRG resident cells were profiled using multiplex arrays to test lysates from four VZV-infected DRG recovered at peak replication, 14 days after infection, and from two mock-infected xenografts (injected with an equal number of uninfected fibroblasts). Concentrations of 18 human cell proteins were significantly increased in VZV-infected DRG lysates, one was significantly decreased, and 33 human proteins were unchanged at 14 days (Fig 8 and S1 Fig and S2 Fig). IFN-alpha, IFN-gamma and IFN-induced proteins IP10/CXCL10 and monocyte-chemoattracting protein MCP-1/CCL2 were significantly increased (2.5, 2.8 20.0 and 7.7-fold, respectively) in VZV-infected DRG. These cytokines are upregulated in peripheral neuroinflammatory responses and in rodent models of neuropathic pain [31,32]. IL-1-alpha, IL-6 and IL-8 and RANTES were also significantly increased (13.1, 7.9, 16.2 and 11.3-fold, respectively); these pro-inflammatory cytokines contribute to inflammatory hypernocicepetion [33–35]. Conversely, TGF-beta, a potent anti-inflammatory cytokine with pleotropic regulatory effects, was also increased (4.4-fold). TGF-beta negatively regulates hepatocyte-growth factor (HGF) which was decreased 2.9 fold, and positively regulates the prosurvival cytokines IL-2 [36] and NGF [20]. Overall, VZV infection elicited dramatic changes in the DRG cytokine milieu, inducing proteins that damage neurons along with some neuroprotective factors, in the absence of adaptive T cell immunity, which is lacking in SCID mice.

## Discussion

**Fig 9:**
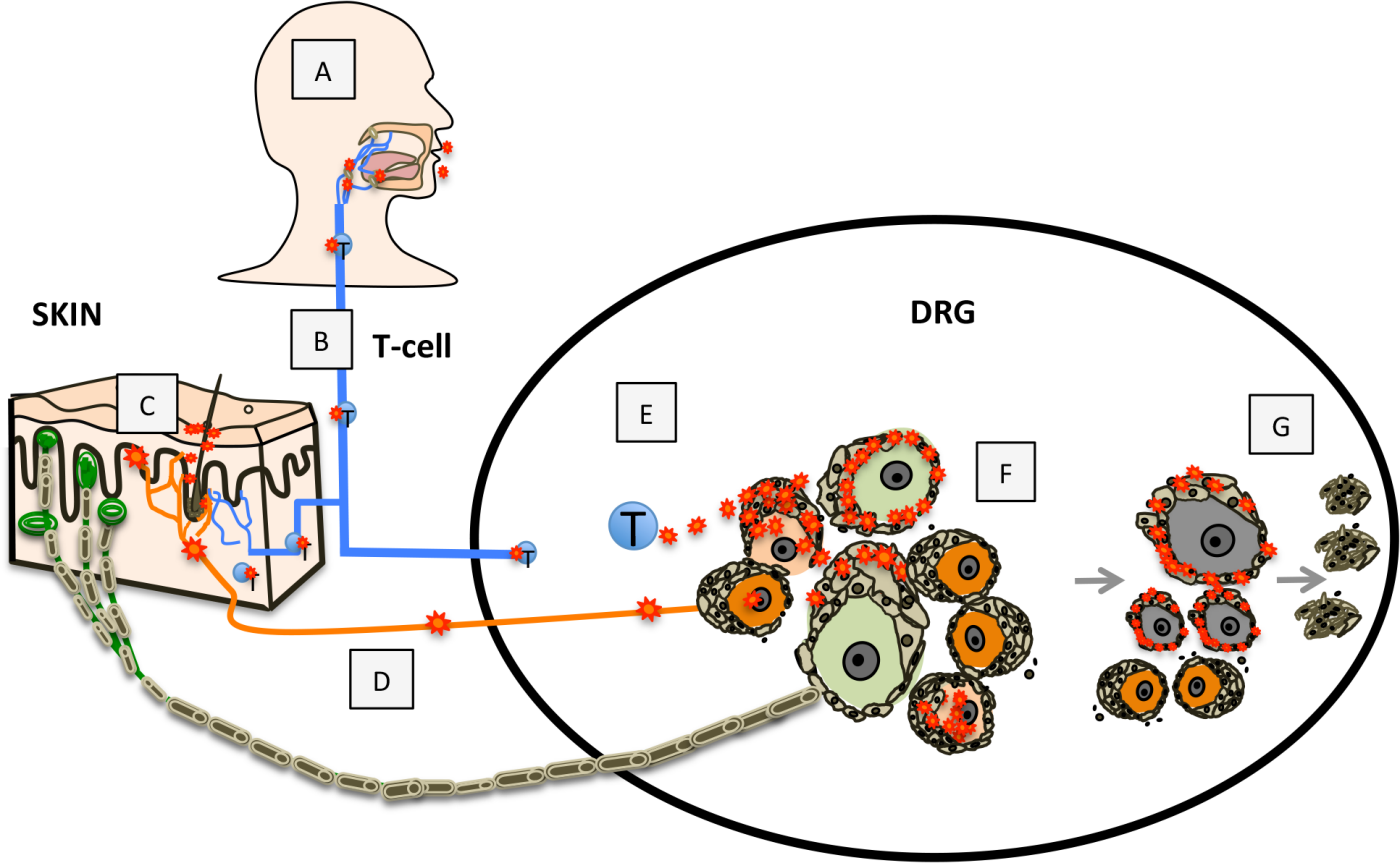
Model for VZV infection of DRG. Primary VZV infection (A) is initiated in respiratory epithelial cells followed by transfer to T cells in tonsils and other regional lymphoid tissue (B) enabling T-cell mediated spread to skin (C) and subsequently to DRG neurons by retrograde axonal transport (D), or T-cell mediated spread to DRG (E), which facilitates SGC infection. VZV gains access to both nociceptive (small, orange) and mechanoreceptive (large, green) neuron cell bodies by either route; however, replication is severely restricted in mechanoreceptive neurons. If neuronal replication is uncontrolled, infection of SGC facilitates contiguous spread to neighboring NSC (F). Contiguous spread with viral transfer into new neuronal cell bodies amplifies the opportunities for VZV delivery to skin sites of replication. Over time, the consequences of VZV infection in SGC contributes to neuronal cell loss, which is indicated by satellite cell microproliferations, referred to as nodules of Nageotte (G).

These observations demonstrating neuronal subtype specificity of VZV replication and the role of SGC in viral spread within ganglia provide new insights about mechanisms of VZV neuropathogenesis and zoster-related neuropathology, as modeled in Fig 9. In the human host, primary VZV infection begins in respiratory epithelial cells, followed by viral transfer into T cells in tonsils and other local lymph nodes (Fig 9A) and trafficking of the infected T cells to the target cells in skin or spinal ganglia that support viral replication (Fig 9B) [10,37]. VZV replication in skin allows virions to enter the termini of neuronal axons with free nerve endings, which are extensive around hair follicles (Fig 9C) and epidermal stem cells lining the follicles are highly permissive for VZV [37]. By analogy with other alphaherpesviruses, the retrograde axonal transport machinery is presumed to move VZ virions to the cell bodies of sensory neurons where lytic infection or latency ensues (Fig 9D). In contrast, when VZV is transported into DRG and released from infected T cells, the architecture of the neuron-satellite cell complex means that infection is most likely initiated in SGC prior to VZV reaching the neuron cell body (Fig 9E and 9F).

In the natural host, tight control of ganglion infection is predicted during primary VZV infection or when VZV reactivates from latency through mechanisms of gene silencing and intrinsic antiviral responses of neurons and SGC [8,38]. In the healthy individual, innate inhibition is reinforced by VZV-specific T cells, that are detected ~72 hours after varicella onset [39] and expand to high frequencies during herpes zoster [1]. However, if neuronal replication is not controlled, viral particles may cross the small gap between neuronal and SGC cell membranes, initiating infection of encapsulating SGC. Since NSC are closely packed within sensory ganglia, VZV can then spread to SGC that surround adjacent neurons (Fig 9F). The potential for productive infection of neurons and neuron-SGC spread during primary VZV infection is supported by clinical observations that individuals with varicella may also have a dermatomal zosteriform rash. During VZV reactivation, contiguous NSC-NSC spread with viral transfer into new neuronal cell bodies has the potential to amplify VZV delivery to skin sites of replication, thereby increasing the extent of the dermatomal rash and enhancing transmission to susceptible individuals. At the same time, virus produced in new skin lesions generates opportunities for virion entry into other axons, allowing the virus to ‘colonize’ more neurons by retrograde transport during reactivation.

Profiling VZV protein expression in DRG neurons showed that VZV reaches RT97+ neuronal cell bodies within DRG xenografts but productive replication was restricted. Neurons expressing this marker were resistant to the virus even when encapsulating SGC were infected. Importantly, this discovery was possible using DRG maintained longterm in SCID mice because neuronal differentiation occurred, subtype heterogeneity was established with the proportions of the two major neuronal subtypes being equivalent to those found in adult human ganglia [5] and SGC retained the capacity to support neurons.

Since SGC are critical for maintaining neuronal homeostasis, loss of SGC function as a consequence of VZV infection would be predicted to affect the survival of both nociceptive and mechanoreceptive neurons. Notably, even though RT97+ neurons had the capacity to restrict VZV replication, neurons expressing this marker showed selective loss relative to peripherin+ neurons at later times in DRG infection when contiguous spread was extensive. Quantitative studies demonstrate that NSC of large-diameter neurons have more SGC, suggesting they are more dependent on SGC-mediated metabolic support than small diameter neurons [40,41]. Small diameter neurons also exhibit a higher resistance to toxins, such as mercury [42]. Alternatively, loss of RT97+ neurons could be a consequence of abortive VZV infection. VZV IE63 expression, which is blocked in RT97+ neurons, has been shown to suppress apoptosis in VZV-infected neuronal cultures [43]. Neuronal destruction is associated with SGC-mediated neuronophagia, which refers to the scavenging by SGC of cell debris resulting from neuronal cell death, and is detected by formation of Nageotte nodules or SGC hypertrophy where neuronal cell loss has occurred [44]. These structures were observed in VZV-infected DRG at late stages of infection, when RT97+ neurons were largely absent (Fig 9G). We propose that the cytopathic response to abortive infection in mechanoreceptive neurons, loss of SGC function or both may explain some sequelae often associated with herpes zoster and PHN, such as mechanical allodynia. As a correlate, clinical studies have demonstrated decreased cutaneous nerve innervation density in biopsies of allodynic skin from PHN patients, indicating axonal loss [45,46], keeping in mind that peripheral nerve degeneration is only one of the pathological features of PHN.

The restriction of VZV replication in RT97+ neurons was incomplete, in that 16.9% of IE63 expressing neurons express the RT97 marker, suggesting that some RT97+ neurons may be more permissive than others. Of interest, HSV-1 and HSV-2 have been found to have subtype specificity in murine ganglia with HSV-1 exhibiting restriction in large diameter neurons immunoreactive for the rodent-specific A5 antibody [46]. Cutaneous RT97+ mechanoreceptive neurons can be further subclassified by the nature of their axonal projections to the spinal cord and specialized terminations in the skin, which transmit information to the CNS about touch, pressure, vibration and tension through Meissner’s, Pacinian, Merkel and Ruffini-type receptors, respectively. At present, classification of neuronal subtypes using established makers is difficult because the staining signal from the reagents overlaps in ganglion sections [5,47], and inability to distinguish functional aspects and the transcriptional state of the neuron. A more precise identification of the non-permissive neuronal subtype(s) may be possible with alternate methods, such as single-cell RNA sequencing of neurons [48].

Despite the entry of VZV particles into the RT97+ subtype, VZV gene expression was blocked after nuclear expression of ORF61 and IE62 and before IE63 production. Newly synthesized ORF61 and IE62 are detected before viral DNA synthesis and IE63 is expressed several hours after IE62 and ORF61, at the same time as nascent viral DNA synthesis [26]. Notably, VZV replication was blocked in RT97+ neurons even though IE62 is the major viral transactivator [28] and IE62 and ORF61 both have potent functions in inhibiting IFN-dependent innate defenses [27,29,49]. In addition to anti-apoptotic functions, IE63 contributes to the capacity of VZV to overcome IFN-alpha mediated innate immune responses [50]. While observations in VZV-infected DRG suggest that these IE63 functions are not required to interfere with infection in RT97+ neurons, they may be important in peripherin+ neurons.

Profiling the cell factors that were upregulated in VZV-infected DRG and analyzing these responses in the context of their known functions indicated that VZV infection induced marked pro-inflammatory responses, as well as proteins of the IFN pathway and neuroprotective responses. The induction of MCP-1/CCL2, IL-1alpha and RANTES and other factors associated with prolonged and sensitized nociception, suggest a link between SGC and neuronal responses to VZV infection and inflammatory sensitization. While inflammatory cytokines were increased, DRG infection was also associated with increased TGF-beta and TGF-beta-related cytokines. Low TGF-beta secretion is associated with healthy neurons [51] and intrathecal infusion of TGF-beta attenuates nerve injury-induced neuropathic pain in rodent models [51,52]. Induction of IP10/CXCL10 in DRG was consistent with IP10 expression in ganglion neurons of zoster patients [53]. It should be noted that, in addition to neurons on SGC, DRG xenografts also contain Schwann cells and fibroblasts and other supportive cells which may release cell factors detected using human-specific antibodies. Recently, the capacity of the human enkephalin protein to modulate nocifensive behaviors in the rat model of VZV neuropathic pain was shown, suggesting that effects of some human neurotoxic and neuroprotective cell factors identified in the DRG model could be explored with this approach [54].

These findings in the DRG model of VZV neuropathogenesis are medically relevant for an improved understanding of herpes zoster and PHN. The pathophysiology of zoster-related neuropathic pain has been attributed to various mechanisms including central sensitization, inflammatory sensitization and biochemical changes in neurons [55,56]. Our experiments suggest that without an early and robust host response to herpes zoster, a VZV-induced gliopathic component mediated by the SGC response to VZV infection and a possible neuron-subtype specific component triggered by nonproductive infection of mechanoreceptive neurons, may contribute to changes associated with PHN. Pathological changes observed in the DRG model reproduce those reported in human cadaver DRG from patients with PHN, including fibrotic changes, axonal degeneration, and loss of large myelinated nerve fibers [57]. Future studies in this model are warranted to determine the molecular mechanism(s) underlying restricted tropism for RT97+ mechanoreceptive neurons and may open avenues to developing improved therapeutic approaches to VZV-related neuropathic pain.

## Methods

### Ethics statement

NIH guidelines for housing and care of laboratory animals were followed (Animal Welfare Assurance #A3213-01), and Institutional Animal Care and Use Committee (IACUC) review of research involving animals was performed, and procedures were approved by the Stanford University Administrative Panel on Laboratory Animal Care (Protocol ID#11130). Use of fetal material has been reviewed by the Stanford University Administrative Panel on Human Subjects in Medical Research and the scope of use does not meet the criteria for research involving human subjects. Anonymized fetal material is provided by the non-profit tissue supply organization Advanced Bioscience Resources, Inc. (ABR) in accordance with applicable federal and state regulations.

### Construction and VZV infection of SCID DRG xenografts

A single human DRG, gestational age 18–22 weeks (~1–2 mm^3^) with attached dorsal root was inserted under the left renal capsule of a sedated six-week old male C.B.-17 *scid/scid* mouse (Taconic Farms, Germantown, NY). DRG were surgically exposed and inoculated by a single direct injection of 10 microliters containing VZV-infected fibroblasts or VZV-infected tonsil T-cells (recombinant parent Oka strain) into the DRG xenograft using a 30-gauge needle. DRG xenografts were recovered at designated timepoints from euthanized mice, fixed with 4% paraformaldehyde, and processed for histological analysis. T cells were isolated from dissociated tonsil tissues obtained via elective tonsillectomy, the T cell fraction was enriched using a nylon wool column, infected by co-culture with a VZV-infected adherent cell monolayer for 72 hours [37], and then 10 microliters of 8x10^4^ VZV-infected T cells (1067 PFU), injected into DRG xenografts engrafted for 8 months, and recovered at 14 and 21 days after infection. Inoculum titers were determined as previously described [10].

### Analysis of tissue sections

3–5 infected DRG xenografts were recovered at each time point, paraffin embedded, and the tissue block was serially sectioned (10 micron thickness) to generating ~150 slides/2 sections per slide. Every 20^th^ slide was stained with hematoxylin and eosin (H&E) to assess cytopathology and locate zones containing clusters of nerve cell bodies. Sequential tissue sections that exhibited cytopathic effect were examined by immunostaining with antibodies to VZV or cellular proteins or VZV DNA *in situ* hybridization (ISH). For each staining condition, slides prepared without the primary antibody and with sections from an uninfected DRG and a known positive DRG were examined in parallel. Enzyme immunohistochemistry and VZV DNA *in situ* hybridization was performed as previously described [10]. For confocal analysis, slides were examined using an AxioPlan 2 LSM 510 microscope (Zeiss, New York, NY). Images were scanned at 1024 x 1024 pixels, 8-frame averaging minimum, and a pinhole size of 1 airy unit. Some images were cropped in Adobe Photoshop. All non-linear adjustments are described in the figure legends. Staining experiments were performed on multiple tissues sections (6–10 sections) for each DRG. For assessment of VZV protein localization and quantitative analysis of neuronal subtype, at least 20 fields, each with a minimum of 50 neurons were examined. Only neurons in which the neuronal nucleus was clearly visible were included in the quantitation for ORF61 and IE62, which are predominantly nuclear proteins.

### Antibodies

The primary VZV antibodies were anti-VZV human polyclonal antibody (1:500 dilution, Ig purified GK serum), rabbit polyclonal antibodies against IE62 (1:200 dilution; provided by Paul Kinchington, University of Pittsburgh), IE63 (1:400 dilution provided by William Ruyechan, University of Buffalo), ORF61 (1:50 dilution, provided by Saul Silverstein, Columbia University) and ORF23 (1:400 dilution), generated in the Arvin laboratory and mouse anti-IE63 (1:800 dilution, provided by Catherine Sadzot-Delvaux, University of Liège). Anti-VZV gE (1:400 dilution) and anti-VZV IE62 (1:400 dilution) are available from Invitrogen, Temecula, CA, and anti-PML (1:400) is available from Santa Cruz Biotechnology, Santa Cruz, CA. Neuronal antibodies included anti-RT97 (1:200, Abcam #178589), anti-peripherin (1:400, Abcam #ab4666), anti-NCAM (1:400, Invitrogen #07–5603), anti-synaptophysin (1:100, Dako #A0010), anti-PML (1:200, Abcam #96051). Antigen retrieval using the citrate buffer/pressure cooker method was performed prior to staining. Secondary antibodies for confocal analysis were Alexa-Fluor 488 (green staining) and Alexa-Fluor 594 (red staining) (Invitrogen, Temecula, CA).

### Multiplex cytokine array

Multiplex immunoassay to measure cytokine levels was performed on whole tissue lysates from 2 mock infected and 4 VZV-infected DRG xenografts recovered at 14 days after infection (rOka strain, 1000 PFU). DRG were homogenized in 300 microliteres cold lysis buffer (Procarta Lysis Buffer), clarified by centrifugation (14000 RPM, 10 min., 4°C), normalized for protein concentration (Biorad DC Protein Assay), and frozen at -80°C until the assay was performed. Each DRG yielded ~30–35 mg of tissue. Lysates contained both intracellular and extracellular (secreted) cytokines. 25 microliters of lysates were added to wells in a multiplex 50-bead array in duplicate wells. Lysis buffer alone established background levels; normal serum was run as a control. Cytokine levels in pg/ml were determined using the Luminex 200 IS System array reader (Luminex) and analyzed using software provided by the manufacturer. Recombinant cytokines were used to establish standard curves, maximize sensitivity and establish the dynamic range of the assay. The read-out is based on sample incubation with fluorescent microspheres conjugated with monoclonal antibodies specific to target proteins. Statistical analyses were performed using GraphPad Prism version 6.0. Reads below the standard curve were assigned the lowest readable value. Values in duplicate wells were averaged, outliers were expunged based on Grubb’s criteria (p <0.05), and T tests determined significance at p <0.05.

## Supporting Information

S1 Fig Induction of cellular factors in VZV-infected DRG. Cellular factors made by SGC and other DRG resident cells were profiled in DRG lysates. Shown here are the 19 cytokines that were statistically increased (N = 18, ttest <0.05) or decreased (N = 1, ttest <0.05), grouped by role (A) interferon-regulated, (B) pro-inflammatory and (C) neuroprotective cytokines. Mock infected, white bars; VZV-infected, black bars. Cytokines (N = 32) that did not meet significant criteria are shown in S2 Fig. Multiplex immunoassay to measure cytokine levels was performed on whole tissue lysates. DRG were homogenized in 300 microliters cold lysis buffer (Procarta Lysis Buffer), clarified by centrifugation (14000 RPM, 10 min., 4°C), normalized for protein concentration (Biorad DC Protein Assay), and frozen at -80°C until the assay was performed. Each DRG yielded ~30–35 mg of tissue. Lysates contained both intracellular and extracellular (secreted) cytokines. 25 microliters of lysates were added to wells in a multiplex 50-bead array in duplicate wells. Lysis buffer alone established background levels; normal serum was run as a control. Cytokine levels in pg/ml were determined using the Luminex 200 IS System array reader (Luminex) and analyzed using software provided by the manufacturer. Recombinant cytokines were used to establish standard curves, maximize sensitivity and establish the dynamic range of the assay. The read-out is based on sample incubation with fluorescent microspheres conjugated with monoclonal antibodies specific to target proteins. Statistical analyses were performed using GraphPad Prism version 6.0. Reads below the standard curve were assigned the lowest readable value. Values in duplicate wells were averaged, outliers were expunged based on Grubb’s criteria (p <0.05), and T tests determined significance at p <0.05.
(PDF)

S2 Fig Additional cytokine array dataset. Cellular factors made by SGC and other DRG resident cells were profiled in DRG lysates. Shown here are the 32 cytokines that were unchanged (did not meet significance by ttest compared with mock-infected). Statistical analyses were performed using GraphPad Prism version 6.0. Mock infected, white bars; VZV-infected, black bars. Cytokines (N = 19) that did meet significant criteria are shown in S1 Fig. Methods are detailed in S1 Fig.
(PDF)
