# Supplementary material for: Neuronal Subtype and Satellite Cell Tropism Are Determinants of Varicella-Zoster Virus Virulence in Human Dorsal Root Ganglia Xenografts In Vivo
Source: PLoS Pathog. 2015 Jun 19;11(6):e1004989. doi: 10.1371/journal.ppat.1004989 (PMC4474629; doi:10.1371/journal.ppat.1004989)
Supplement: S1 Fig — Cellular factors made by SGC and other DRG resident cells were profiled in DRG lysates. Shown here are the 19 cytokines that were statistically increased (N = 18, ttest <0.05) or decreased (N = 1, ttest <0.05), grouped by role (A) interferon-regulated, (B) pro-inflammatory and (C) neuroprotective cytokines. Mock infected, white bars; VZV-infected, black bars. Cytokines (N = 32) that did not meet significant criteria are shown in S2 Fig. Multiplex immunoassay to measure cytokine levels was performed on whole tissue lysates. DRG were homogenized in 300 microliters cold lysis buffer (Procarta Lysis Buffer), clarified by centrifugation (14000 RPM, 10 min., 4°C), normalized for protein concentration (Biorad DC Protein Assay), and frozen at -80°C until the assay was performed. Each DRG yielded ~30–35 mg of tissue. Lysates contained both intracellular and extracellular (secreted) cytokines. 25 microliters of lysates were added to wells in a multiplex 50-bead array in duplicate wells. Lysis buffer alone established background levels; normal serum was run as a control. Cytokine levels in pg/ml were determined using the Luminex 200 IS System array reader (Luminex) and analyzed using software provided by the manufacturer. Recombinant cytokines were used to establish standard curves, maximize sensitivity and establish the dynamic range of the assay. The read-out is based on sample incubation with fluorescent microspheres conjugated with monoclonal antibodies specific to target proteins. Statistical analyses were performed using GraphPad Prism version 6.0. Reads below the standard curve were assigned the lowest readable value. Values in duplicate wells were averaged, outliers were expunged based on Grubb’s criteria (p <0.05), and T tests determined significance at p <0.05. (PDF) [file ppat.1004989.s001.pdf]

SI Fig. Induction of cellular factors in VZV-infected DRG

A. Interferon-regulated cytokines

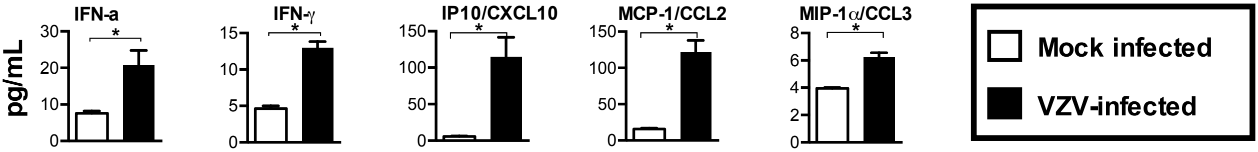

B. Pro-inflammatory cytokines

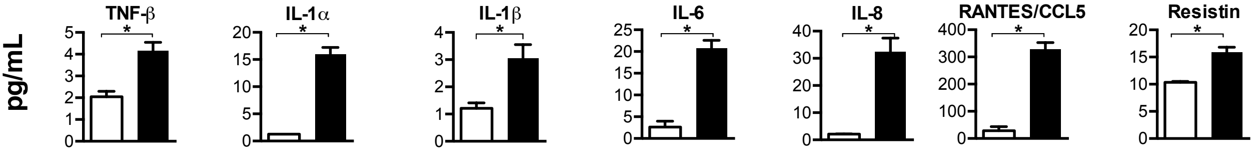

C. Neuroprotective cytokines

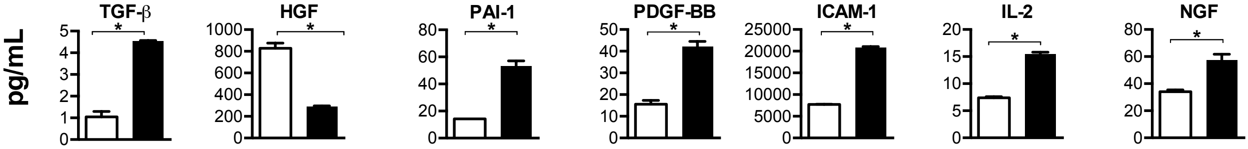

Cellular factors made by SGC and other DRG resident cells were profiled in DRG lysates. Shown here are the 19 cytokines that were statistically increased (N=18, ttest <0.05) or decreased (N=1, ttest <0.05), grouped by role (A) interferon-regulated, (B) pro-inflammatory and (C) neuroprotective cytokines. Mock infected, white bars; VZV-infected, black bars. Cytokines (N=32) that did not meet significant criteria are shown in **S2 Fig**.

Multiplex immunoassay to measure cytokine levels was performed on whole tissue lysates. DRG were homogenized in 300 ml cold lysis buffer (Procarta Lysis Buffer), clarified by centrifugation (14000 RPM, 10 min., 4°C), normalized for protein concentration (Biorad DC Protein Assay), and frozen at -80°C until the assay was performed. Each DRG yielded ~30-35 mg of tissue. Lysates contained both intracellular and extracellular (secreted) cytokines. 25 ul of lysates were added to wells in a multiplex 50-bead array in duplicate wells. Lysis buffer alone established background levels; normal serum was run as a control. Cytokine levels in pg/ml were determined using the Luminex 200 IS System array reader (Luminex) and analyzed using software provided by the manufacturer. Recombinant cytokines were used to establish standard curves, maximize sensitivity and establish the dynamic range of the assay. The read-out is based on sample incubation with fluorescent microspheres conjugated with monoclonal antibodies specific to target proteins. Statistical analyses were performed using GraphPad Prism version 6.0. Reads below the standard curve were assigned the lowest readable value. Values in duplicate wells were averaged, outliers were expunged based on Grubb's criteria ($p < 0.05$), and T tests determined significance at $p < 0.05$.
