# Supplementary material for: Neuronal Subtype and Satellite Cell Tropism Are Determinants of Varicella-Zoster Virus Virulence in Human Dorsal Root Ganglia Xenografts In Vivo
Source: PLoS Pathog. 2015 Jun 19;11(6):e1004989. doi: 10.1371/journal.ppat.1004989 (PMC4474629; doi:10.1371/journal.ppat.1004989)
Supplement: S2 Fig — Cellular factors made by SGC and other DRG resident cells were profiled in DRG lysates. Shown here are the 32 cytokines that were unchanged (did not meet significance by ttest compared with mock-infected). Statistical analyses were performed using GraphPad Prism version 6.0. Mock infected, white bars; VZV-infected, black bars. Cytokines (N = 19) that did meet significant criteria are shown in S1 Fig. Methods are detailed in S1 Fig. (PDF) [file ppat.1004989.s002.pdf]

S2 Fig. Additional cytokine array dataset

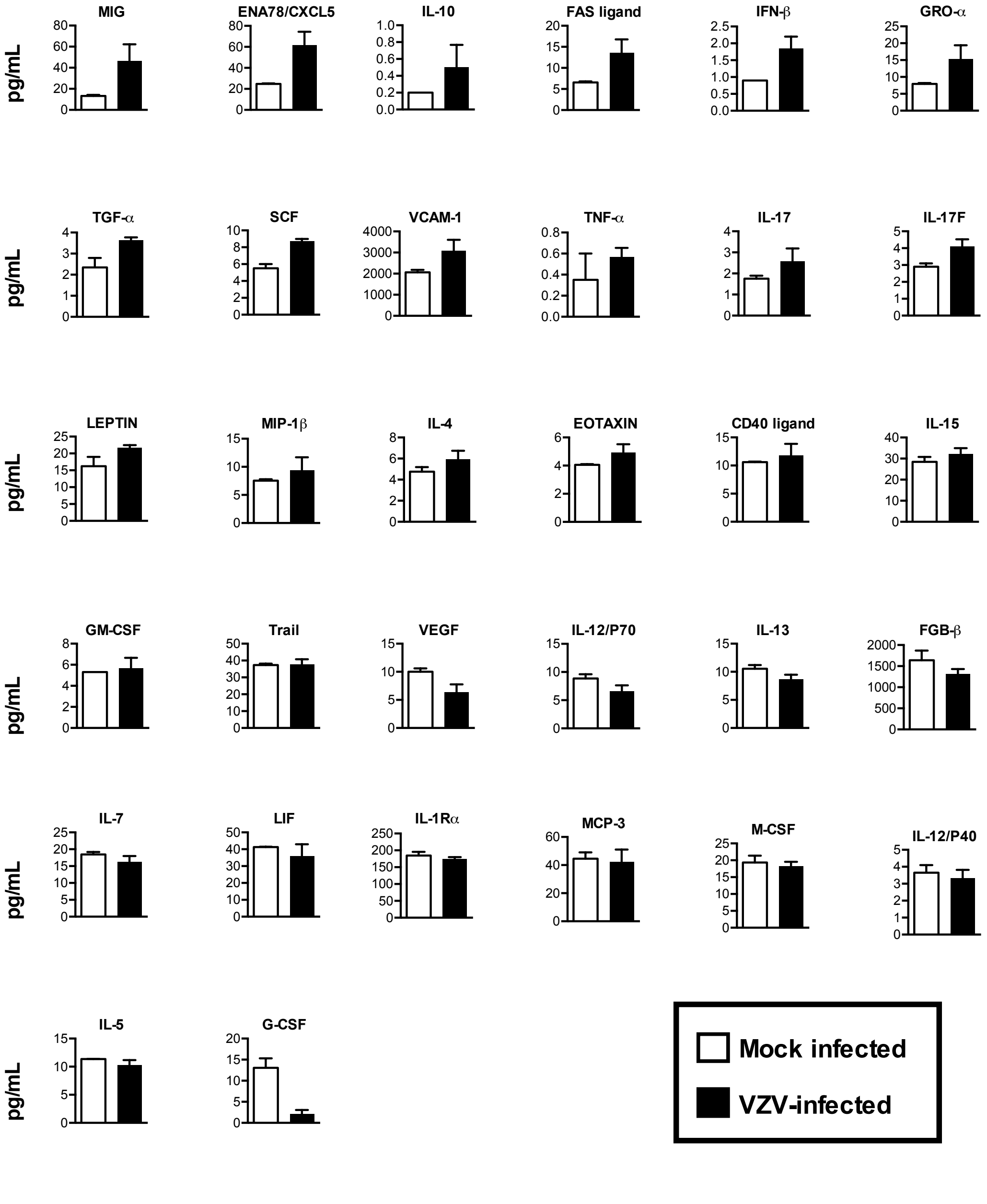

Cellular factors made by SGC and other DRG resident cells were profiled in DRG lysates. Shown here are the 32 cytokines that were unchanged (did not meet significance by ttest compared with mock-infected). Statistical analyses were performed using GraphPad Prism version 6.0. Mock infected, white bars; VZV-infected, black bars. Cytokines (N=19) that did meet significant criteria are shown in **S1 Fig**. Methods are detailed in **S1 Fig**.
